# Supplementary material for: Improving Fundamental Movement Skills during Early Childhood: An Intervention Mapping Approach
Source: Children (Basel). 2023 Jun 2;10(6):1004. doi: 10.3390/children10061004 (PMC10297377; doi:10.3390/children10061004)
Supplement: Supplementary file 1 [file children-10-01004-s001.zip › children-2357852-supplementary.pdf]

Supplementary materials

Table S1 Planning group Focus group themes and comments Step 1

Step one areas	Researchers' themes and quotes	Parents themes and quotes	Teachers', coaches, SLT themes and quotes
Who's problem is it?		Family and parents, school	Headteachers, teachers ('It depends on confidence'), families
Who is the Problem affecting?	'Children are arriving at school inactive'		'Obesity is um, a ridiculously growing problem in this country'
Individual behaviours causing the problem	Children are inactive at home<br>Children are not developing their motor skills	Culture and mentality<br>'kids aren't as bothered' about PA<br>High screen time and use of technology	Use of technology 'sit and look into iPads and Xboxes'<br>'Children having more access to, to, tablets, computers, culturally things have changed in terms of playing outside'
Environment conditions causing the problem	Teachers/staff 'undertrained' and 'not enthusiastic'<br>Individual 'school level won't create change' in the whole system<br>Previous Intervention 'too prescriptive'	Restriction of free play<br>Pick up and drop off culture<br>Lack of facilities for PA and 'things not being open'<br>'School provision [for PA] isn't great'	'I think if the school's got good resources… and we had funding'<br>'Play led but not enough specific skills'<br>families: 'they won't do anything that upsets their child'<br>'There isn't really much emphasis on like developing skills'
Determinants of these behaviours	'Self-efficacy for the children would be to have a go at a new skill'<br>Deprivation<br>Low confidence and self-efficacy of teachers<br>School philosophy	Education of parents and lack of confidence- 'be careful' culture<br>Having other children to play with<br>Money and deprivation 'after school clubs aren't free anymore'	'[Children] don't have that opportunity at home because their parents, they can't afford to take them to do extra-curricular things'<br>'I don't believe that there's adequate PE time'
Social assets	Family, schools can create 'awareness'	Football and multicurricular clubs<br>'Play with peers and older siblings'	
Information assets	Teachers could be if 'enthusiastic'	Word of mouth- 'other parents'	Teachers- depends on confidence levels<br>'A really good resource and you can dip into it and out'
Policy Assets	National governing bodies<br>Change needs to come from 'policy level to get widespread effects and there needs to be an incentive to be involved'	'Schools can vary quite dramatically in their provision, time and access to PA'	'A huge culture shift I think to overcome some of that'
Environment Assets	Schools are 'equitable and accessible to all children'	Active transport to school<br>Free play outside 'in good weather'<br>PE 'part of the school day'	'More active lessons, much more standing up and moving round and talking to other people'<br>'To be outside and to be doing things outside'

PA= physical activity, PE=Physical education

Table S2. Potential desired outcomes

**Behavioural:**
Increase the amount of time EYFS children engage in FMS practice at school
Increase the amount of time EYFS children spend doing PA at home and at school
Reduce the sedentary time of EYFS children at home and at school
**Environmental:**
Increase the provision of FMS delivery by 25% at the EYFS in schools
Increase the number of schools developing an EYFS PE curriculum
Increase the number of teachers receiving PE and PA training

Table S3 Matrices of change: Behavioural Outcome

	**Behavioural Outcome: 1.0 Increase the number of EYFS children engaging in FMS practice**			
**Performance objective**	**Change Objectives for Personal Determinants**			
	**Mastery Level of FMS**	**Self-efficacy/physical self-concept**	**Perceived Norms and enjoyment**	**Knowledge**
**PO.1.1 Increase the % of children spending 10% more time doing PA in school each week**	**ML.1.1.** Children demonstrate greater mastery of FMS to use in PA in free play situations and environments	**SE.1.1.** Children demonstrate confidence when performing PA and chose multiple forms of PA	**PN.1.1.** Children enjoy and perform increased levels of total PA	**K1.1.** Children can describe an FMS based game learnt at school
**PO.1.2 Increase the % of children taking part in structured and unstructured FMS related activities each week**	**ML.1.2.** Children demonstrate the FMS ability to participate in physical FMS games each week	**SE.1.2.** Children demonstrate confident use of skill within 2 or more game environments	**PN.1.2.** Children chose to participate in FMS based games/activities when offered during free choice play opportunities	**K.1.2.** Children know what different kinds of FMS are through physical and classroom-based activity
**PO.1.3 Increase the % of children setting FMS and PA performance goals at school**	**ML.1.3.** Children can continue to master new FMS and PA goals set at school	**SE.1.3.** Children know how to begin to set their own goals e.g., catching a ball 3 times in a row	**PN.1.1.** Children are involved in setting individual goals with their parent/teachers for PA. E.g., walk or scooter to school once a week	**K.1.3.** Children know how to use feedback, can explain their FMS skills and goals set

FMS=fundamental movement skills, PA= physical activity

Table S4 Matrices of change: Environmental Outcome

Performance objective	2.0 Increase the provision of FMS delivery by 20% at the EYFS in schools				
	**Change Objectives for Personal (environmental) Determinants**				
	**Knowledge**	**Social norms**	**Teacher self-efficacy**	**Parental Beliefs**	**Outcome expectations**
**PO.2.1** **Increase the number of teachers facilitating FMS practice in EYFS settings**	**K.2.1.** Teachers know how to implement the provided training sessions and resources. Teachers demonstrate an increased knowledge for facilitating FMS practice in a school setting	**SN.2.1.** Teachers demonstrate that it is important their school community provides FMS practice. Their school provides environments for FMS practice	**TSE.2.1.** Teachers show increased self-confidence to lead sessions with an FMS focus, and know how to give sufficient positive and skill specific feedback, adapted to suit the ability of the child	**PB.2.1.** SLT and Teachers in schools can share information with parents about FMS and benefits behind the programme	**OE.2.1.** Teachers expect that providing better structure in existing and new PA opportunities, will improve children's PA and FMS
**PO.2.2 Increase the number of teachers planning for PE, PA, and FMS in schools**	**K.2.2.** Key agents (teachers and SLTs) know how to be equipped with materials and resources to improve planning, including an adaptable framework	**SN.2.2.** Teachers make give FMS and PA equal status as academic subjects and highlight the PE premium use	**TSE.2.2**. Teacher can show evidence of planning for effective PA and saving time in the process	**PB.2.2.** School/ teachers share information of how PA and FMS can be improved in the home environment and prepare child for PE	**OE.2.2.** Teachers expect to use a framework and improve the quality of current PA by structuring more opportunities to improve PA/FMS
**PO.2.3 Increase the number of teachers aware of the benefits of providing EYFS children with PA opportunities**	**K.2.3.** Teachers can explain why PA for children is important for physical, mental, and social health, in addition to enhancing academic achievement	**SN.2.3.** Teachers can demonstrate the importance of a whole class targets and individual goals to perform a certain amount of PA per week	**TSE.2.3.** Teacher can explain effective ways to engage children in more PA outside of PE and assess their skills	**PB.2.3.** Teacher knows how to share information about the benefits of PA to parents	**OE.2.3.** Teachers expect to allocate more specific times to dedicated PA-twice a week in non-PE subjects

FMS=fundamental movement skills, PA= physical activity, PE= physical education, EYFS= early years foundation stage

Table S5 Planning group Focus group themes and comments Step 3: Task 3.1

Step three areas	Researchers themes and quotes	Parents themes and quotes	Teachers, coaches, SLT themes and quotes	Children themes and ideas
Programme Themes	‘Autonomous activity’<br><br>‘Children enjoy having freedom and ownership of their experiences’<br>Previously experienced: Gruffalo, acting out story books with movements	Dinosaurs, cars/trucks, superheroes, Bluey (many agreed Bluey was a good cartoon role model)<br><br>‘Working together’<br><br>‘We do lots of role play as a family’	‘Progress in, in actually not just sport but in physical um, in physical life’<br><br>‘5-minute movements’<br><br>‘Simple skills’	Superheroes, book characters, cartoons, movie characters (see table A7 for further detail)
Programme Components	Connection between the school and home environment<br><br>Improving ‘teacher self-efficacy’<br><br>‘Hide in fun to improve other skills’ such as numeracy	‘Not instructional activity’<br><br>Competitive elements such as ‘racing’<br><br>‘Progressing through levels’<br><br>‘Physical activity homework, within their existing school framework’<br>‘Education of teachers and head teachers’	‘I think maybe an overarching overview, so where their skill development is, and like a progression of skills document’<br><br>‘Having new ideas and thing put into like the frame[work]’<br><br>‘Having focussed lessons, um, I think will see it improve’	Games based activities for children- ‘parachute’, ‘shark game’, ‘tag football’
Programme Sequence	‘Keeping it simple helps the buy in’<br><br>‘Keeping a connection between the home and school environment’	‘Do stuff they [children] know and has structure’<br>‘I think it should sit within the school framework, slot into regular assessments’	‘Giving them [children] opportunities to practice and then keep teaching them’<br><br>‘The children get different opportunities throughout the week’	
Programme Scope and duration	‘Daily activity helps to keep them involved’<br><br>Create as an ‘enrichment’ activity for the children	School to manage the programme- and ‘let the children be independent in the environment’<br><br>App development- ‘gamification’ of elements	‘If we can teach everyone at the same time its inclusive’<br><br>‘The teachers and the heads want something concrete to be left behind’<br>‘If you give them [teacher] a framework and scaffold and some structure, they’d be happy to carry it on’ and ‘You need to be doing it 3 or 4 times a week’	

Table S6 Planning group Focus group themes and comments Step 3: Task 3.3 measurement of the programme, programme environments and creating awareness

Step three areas	Researchers themes and quotes	Parents themes and quotes	Teachers, coaches, SLT themes and quotes	Children themes and ideas
Measurement of the programme	Logbooks to examine children's physical literacy<br><br>Teacher to report using 'formative feedback'<br><br>'Physical development judgements are part of the EYFS, so working it into that'	'Slot into the school framework' e.g., part of reports home<br><br>'[child has] confidence to take part in a new sport (activity)'<br><br>'Education of teachers and head teachers'<br>'Evaluating the physical and holistic development'	'Sense of achievement for the children'…'a bit of aspiration, they're trying to beat their score'<br><br>'So, they could see progression in themselves, that they were able to do that'<br><br>'We do a thing called impact report… we do it overall as a year group'<br><br>'Regardless of if it's like scientific or if it's you can catch three times rather than two times by the end of the year'	Children stated in their focus groups that PE made them feel: 'happy', 'excited', that they were having 'fun', the programme must achieve this and should be evaluated by teachers
Programme Environments	'Outdoor environments for autonomous activity'<br><br>'Indoor and classroom active breaks'<br><br>Vary the environment to allow adaptation for 'wet play situations'	'Free play environments'<br><br>'Leaving the house' outdoors and unfamiliar environments<br><br>'Social environments with their peers'	'Lots of access to outdoor play and to be able to run and to walk and to ride a bike and to ride tricycles'<br><br>'Open flow through the classroom area and out into the outdoor area'	Playing<br>Outdoors<br>Equipment- climbing frames, parachutes, balls, sandpits, hula hoops<br>Indoors for climbing, dancing
Creating awareness	Highlight the benefits of the programme to the teachers- 'what they're judged on' e.g., improving school readiness of the children	Something 'that physically comes home in a pack, rather than online' (for parents)<br><br>'To get support to do it at home as PA homework'	'Somebody there to disseminate that knowledge and work with them [teachers]'	Non-PA based activities to increase children's awareness of FMS skills (see Table 5.11 for detail)

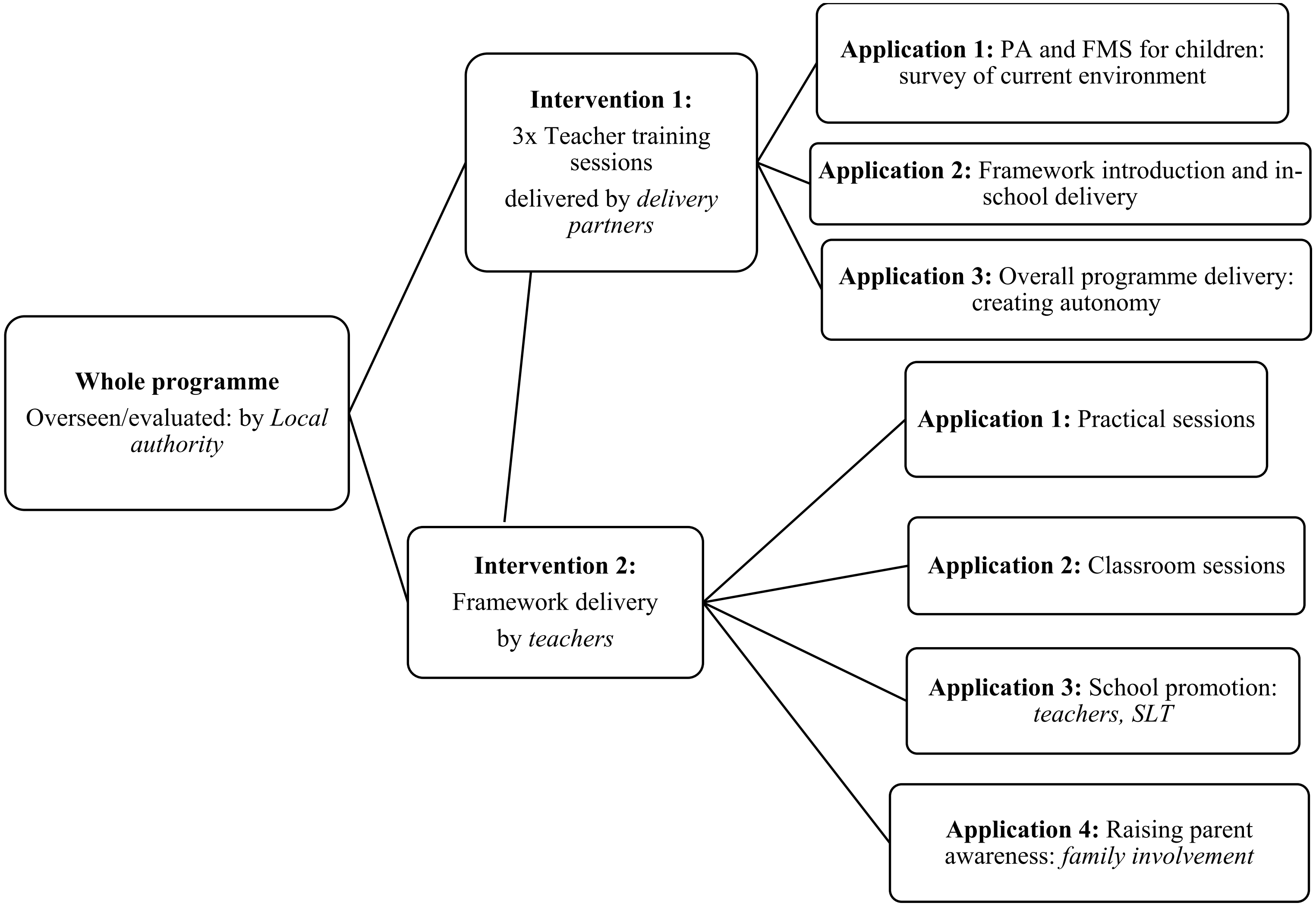

Figure S1. Task 4.1: Programme structure and organisation
FMS=fundamental movement skills, PA= physical activity, SLT= senior leadership team

Table S7 Task 4.3: Focus group themes and answers

Programme design area	Child FG answers	Other FG suggestion
Equipment	Parachutes, balls, sand pit, climbing frames, hula hoops, rackets	
Environments	Outdoors, classroom/hall spaces	'Independent environments', 'getting outside'
Types of activities	Building, 'playing with my friends', 'making up my own games', games with easy-to-understand rules, dancing, football, climbing, running	'Making things up, using their imagination' 'jumping off things, climbing up stuff', games- 'racing each other', 'acting out a story', 'education hiding in fun',
Themes of games/activities	'Shark game', Dinosaurs, Zog, Thomas the Tank Engine, Spiderman, Supergirl, Superman, Paw Patrol, Rapunzel, Harry Potter,	Superheroes, role play, 'family involvement'
Non-physical activities	Reading, literacy, doing puzzles, 'new things'	'Use of an app', 'classroom active breaks'

Figure S2. The FMS School Project logo

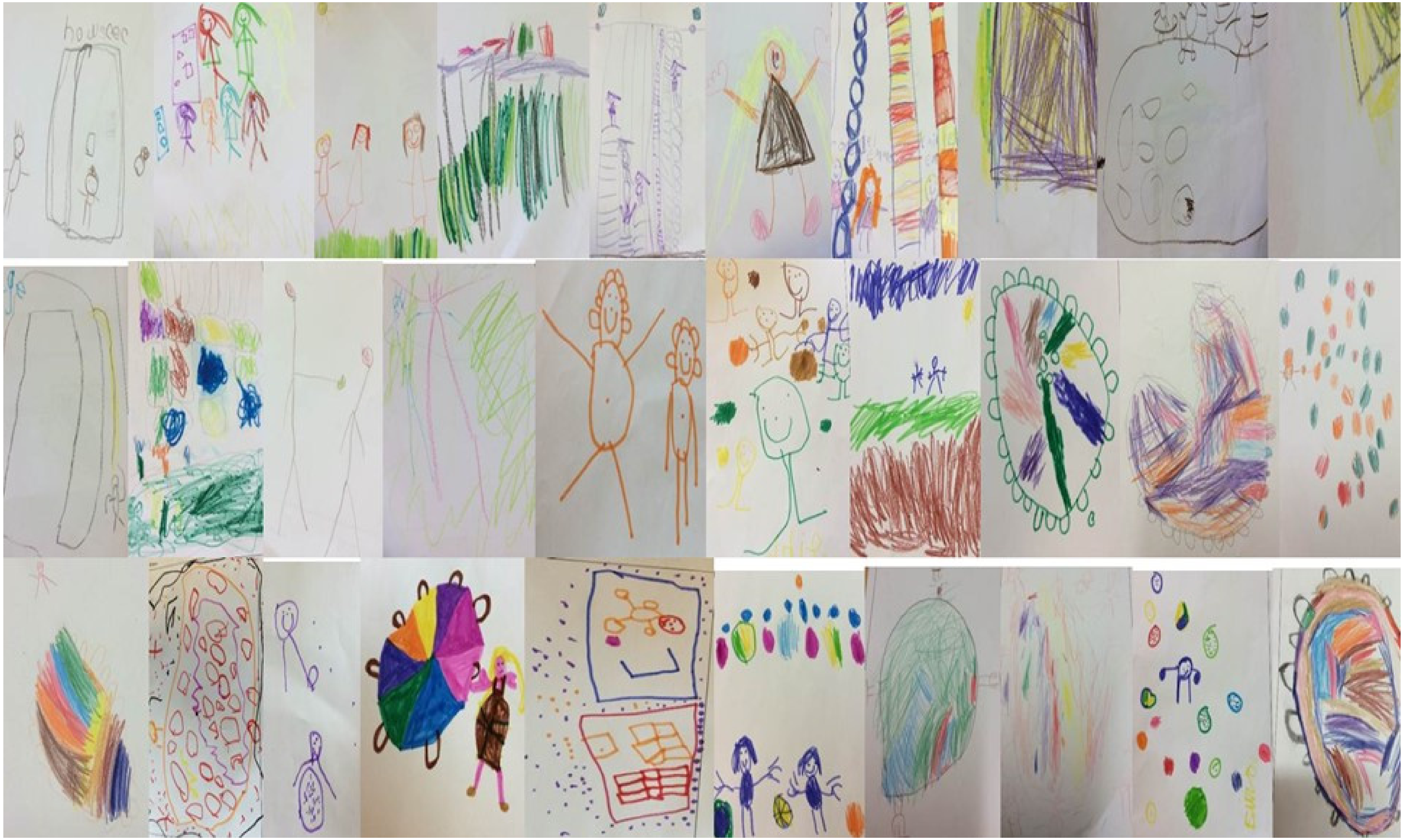

Figure S3. Drawings produced by children in FG1C about their favourite part of physical education.

Supplementary Session S1. Session plan for EYFS children

<table>
<tr>
<td>Time:
35 mins</td>
<td>Aim(s):
1) Practice and begin to develop at least 3 skills (jump, coordination)
2) Discover new pieces of equipment, think of ways to use these objects.
3) Develop our teamwork and listening skills</td>
<td colspan="2">Equipment:
Cones, hoops, woggles, balls, rackets, bibs</td>
</tr>
<tr>
<td rowspan="2">Activities:</td>
<td>1. <u>Fogs and lily pads</u>
▪ Ask the children to show you how a frog would jump- the children should use their imagination to do this. You can also ask the children what noise a frog might make.
▪ Set out spots across the playing area as 'lily pads', ask the children to travel as frogs between the lily pads
▪ Include demonstration of jumping to give the children a visual guide

S- ask children what shape the pond is, get them to think about shapes, make the pond bigger so they must jump further
T- Children could become pond skaters and move across the pond on all fours
E- When a child reaches a lily pad, they must perform a balance/ lily pad could be hoops
P- one big pond or lots of smaller ponds, working in pairs</td>
<td rowspan="2">Skills focus</td>
<td>1. Jumping and locomotor skills</td>
</tr>
<tr>
<td>2. <u>Equipment Discovery</u>
▪ Within the playing area set up a selection of equipment for the children to use, including hoops, woggles, balls (3 different sizes), rackets.
▪ Ask the children to use and play with the equipment however they like e.g., they could make up games or build different structures for example.
▪ Observe how the children use a variety of equipment, how they move e.g., actions like squatting down, the grip they use to hold equipment.

S- use both indoor and outdoor settings, pretend children are in different natural environments.
T- set tasks such as making an obstacle course and others in the class completing it
E- each time this task is used with the children introduce a new piece of equipment or remove a previous piece so that children become more imaginative
P- children can work individually, in pairs, small groups, or as a whole class.</td>
<td>2. Combination and discovery skills</td>
</tr>
</table>

	3. <u>Move through the hoop</u><br>▪ Start with a hoop that is big enough for all the children to fit through<br>▪ The children should stand in a circle holding hands, they must pass the hoop up or over each one of them in the full circle<br><br>S- start as a whole class task in one big group, decrease into smaller groups<br>T-challenge the children to do it faster than they did it the previous time<br>E- use a bigger hoop to start with, as the children get more proficient the size of the hoop can be decreased<br>P- mix the children up and get them to work next to a child they usually don't work with, to help them improve their teamwork		3. Coordination and communication skills
	4.		4.
Formative feedback:	a) recover start of the session by becoming frogs again<br>b) big, middle, or small smiley		

Table S8 Task 5.3: Matrices of change: Dissemination

3.0 Increase the number of delivery partners delivering The FMS School Project training			
**Performance objectives for programme dissemination**	**Change Objectives for Determinants**		
	**Knowledge**	**Skills and self-efficacy**	**Perceived Norms**
**PO.3.1 Increase the number of schools receiving The FMS School Project information**	**K.3.1.** Local authorities know how to disseminate information clearly and effectively about the programme and its components	**SSE.3.1.** Local authorities can confidently disseminate the benefits of the programme to schools in the local area	**PN.3.1.** Local authorities can explain that is its normal to keep in contact with schools about FMS education
**PO.3.2 Increase the number of local authorities using delivery partners for The FMS School Project training**	**K.3.2.** Local authorities know the scope, sequence, theme, and components of the programme	**SSE.3.2.** Local authorities show they can identify the correct delivery partners to provide the programme training in schools	**PN.3.2**. Local authorities can explain it is normal to use this kind of training in schools and for public health improvement

**PO.3.3 Increase the number of local authorities planning to use delivery partner for The FMS School Project training**	**K.3.3.** Local authorities know the benefits of using delivery partners for The FMS School Project training	**SSE.3.3.** Local authorities can plan to employ or identify existing employees as delivery partners	**PN.3.3.** Local authorities can explain that is expected that delivery partners are employed to deliver intervention training

FMS= fundamental movement skills

Table S9 Task 5.3: Matrices of change: implementation and adoption

**4.0 Increase the number of schools delivering The FMS School Project framework**			
**Performance objectives for programme implementation and adoption**	**Change Objectives for Determinants**		
	**Knowledge**	**Skills and self-efficacy**	**Perceived Norms**
**PO.4.1 Increase the number of schools receiving The FMS School Project training**	**K.4.1.** Delivery partners know how to explain the scope, sequence, theme, and components of the programme (training and framework) to teachers	**SSE.4.1.** Delivery partners demonstrate the ability to plan the training delivery and offer support to teachers and SLT in schools	**PN.4.1.** Delivery partners explain that good continued professional development and training of teachers is important for children's development
**PO.4.2 Increase the number of schools planning to receive The FMS School Project training**	**K.4.2.** Delivery partners can explain the training and framework of the programme	**SSE.4.2.** Delivery partners demonstrate how to disseminate training to teachers and SLT in schools	**PN.4.2**. Delivery partners explain good FMS of children at school is the result of sufficient continued professional development and knowledge
**PO.4.3 Increase the number of schools interested in using The FMS School Project training**	**K.3.3.** Delivery partners know how to communicate with schools and SLT about the programme delivery	**SSE.3.3.** Delivery partners can make a case for The FMS School Project training and framework	**PN.3.3.** Delivery partners explain that other schools have successfully implemented The FMS School Project

FMS= fundamental movement skills, SLT= senior leadership team

Table S10 Task 5.3: Matrices of change: maintenance

**5.0 Increase the number of Schools using The FMS School Project framework for more than one school year**			
**Performance objectives for programme maintenance**	**Change Objectives for Determinants**		
	**Knowledge**	**Skills and self-efficacy**	**Perceived Norms**
**PO.5.1 Increase the number of teachers evaluating The FMS School Project framework at the EYFS**	**K.5.1.** Teachers can explain the evaluation methods of the programme to identify success and areas of improvement	**SSE.5.1.** Teachers can demonstrate the ability to evaluate the programme over the whole year, including its individual component.	**PN.5.1.** Teachers can explain schools like ours are providing The FMS Schools Project framework to children at the EYFS
**PO.5.2 Increase the number of SLTs granting the appropriate funds for The FMS School Project**	**K.5.2.** SLT can explain how to use the PE primary funding for The FMS School Project	**SSE.5.2.** SLT can use the appropriate funding for equipment and the training of staff	**PN.5.2**. SLT can explain that successful FMS School Project programmes in their school are a result of the correct funding

**PO.5.3 Increase the number of schools providing the appropriate time for The FMS School Project framework**	**K.5.3.** SLTs and teachers can explain how to use time effectively for FMS development in EYFS children	**SSE.5.3.** SLT and teachers can use timetabling and planning in the school year for The FMS School Project	**PN.5.3.** SLT and teachers can explain that successful FMS programmes have sufficient time within the school year allotted to them

FMS= fundamental movement skills, SLT= senior leadership team, EYFS= early years foundation stage

Table S11 Task 5.3: Matrices of change: maintenance

**6.0 Increase the number of delivery partners delivering The FMS School Project training for more than one school year**			
**Performance objectives for programme maintenance**	**Change Objectives for Determinants**		
	**Knowledge**	**Skills and self-efficacy**	**Perceived Norms**
**PO.6.1 Increase the number of delivery partners/local authorities evaluating The FMS School Project training**	**K.6.1.** Delivery partners and local authorities can explain the evaluation methods of the programme to identify success and areas of improvement	**SSE.6.1.** Local authorities can demonstrate the ability to evaluate the training programme over the whole year	**PN.6.1.** Local authorities can explain areas like ours are providing The FMS Schools Project training to teachers
**PO.6.2 Increase the number of local authorities granting the appropriate funds for The FMS School Project training delivery**	**K.6.2.** Local authorities explain how to allot funding for employment of delivery partners for The FMS School Project training	**SSE.6.2.** Local authorities can use the appropriate funding for employment and training of delivery partner staff	**PN.6.2**. Local authorities can explain that successful FMS School Project programmes in their area are a result of the correct funding
**PO.6.3 Increase the number of local authorities providing the appropriate time for The FMS School Project training**	**K.6.3.** Local authorities can explain how to use time effectively for The FMS School Project training	**SSE.6.3.** Local authorities can allow delivery partners appropriate time to plan, prepare and deliver The FMS School Project training	**PN.6.3.** Local authorities can explain that successful FMS School Project training have sufficient time to train teachers in schools

FMS= fundamental movement skills

Table S12 Step 6: Planning group Focus group themes and comments

**Step 6 areas**	**Researcher's themes and quotes**	**Parents themes and quotes**	**Teachers, coaches, SLT themes and quotes**
Assessment methods	'The methods need to be fun and simple, especially for the children'<br>'Process evaluations and formative feedback'<br>'Logbooks- physical literacy tracking, self-reflection'	'Real-world success measured better using a qualitative questioning approach [for adults]'<br>'Offer children new opportunities and observe the uptake'	'For me to do it [the intervention] and get feedback [from a delivery partner]'

Reporting	‘Schools can use the images from a children’s write, draw, show and tell activity’<br>‘Evidence of grants being used for the programme’<br>Using tools that are being developed by researchers to be implemented in schools.	‘Sit within the school’s existing reporting framework’<br>‘Should be integral and made compulsory’<br>‘Schools reporting, they are using the programme to educate their staff’	‘Having learning outcomes are a big one’<br>‘a clear expectation of what resources would be required…depending on cost’
What should be assessed?	‘How did the people using the intervention find it?’<br>‘The enjoyment and development of the children using the programme’<br>‘parent’s expectations of the programme’	‘The quality of the PE [framework sessions] being provided in school’<br>‘Culture change within schools by the senior leadership team’<br>‘children’s confidence to take part in new sport’	‘The longevity in it’<br>‘PE is one of the lessons that teachers plan the least, so this should be assessed’<br>‘We should also develop our practices [teachers]

PE= physical education

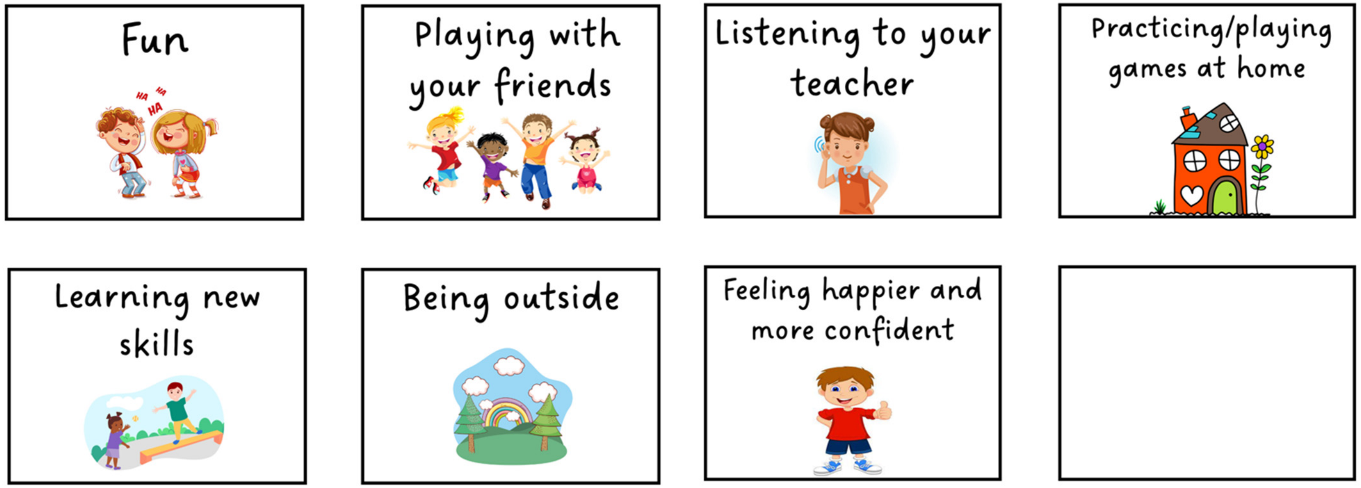

Figure S4. Task 6.4: Child Process Evaluation Card Sort

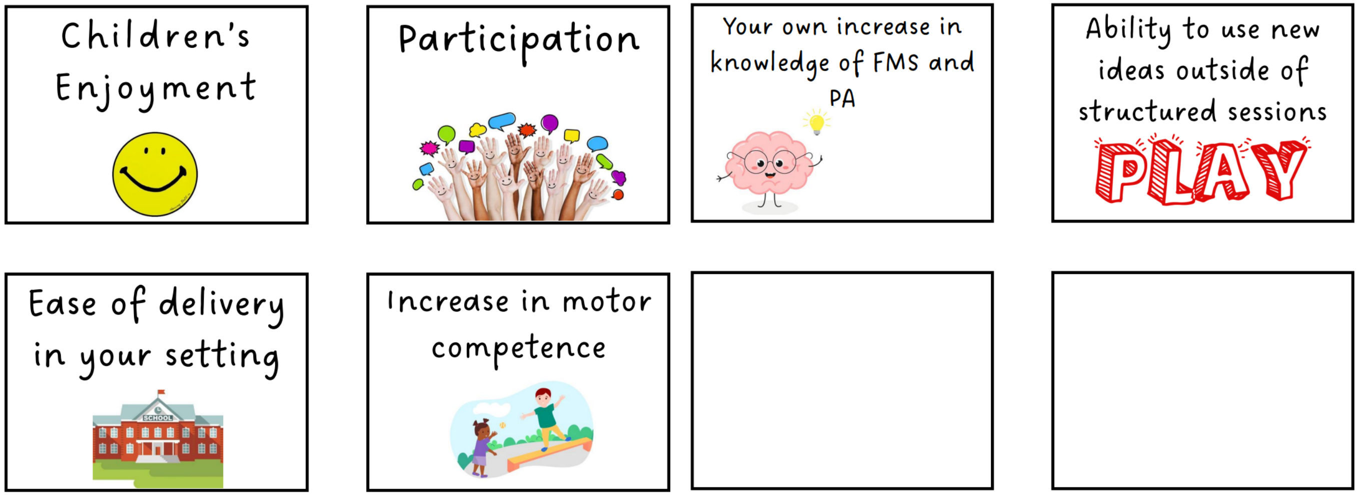

Figure S5. Teacher card sort
